# Identification and Characterization of Oleaginous Yeast Isolated from Kefir and Its Ability to Accumulate Intracellular Fats in Deproteinated Potato Wastewater with Different Carbon Sources

**DOI:** 10.1155/2017/6061042

**Published:** 2017-09-17

**Authors:** Iwona Gientka, Marek Kieliszek, Karolina Jermacz, Stanisław Błażejak

**Affiliations:** Department of Biotechnology, Microbiology and Food Evaluation, Faculty of Food Science, Warsaw University of Life Sciences-SGGW, Nowoursynowska Str. 159c, 02-776 Warsaw, Poland

## Abstract

The search for efficient oleaginous microorganisms, which can be an alternative to fossil fuels and biofuels obtained from oilseed crops, has been going on for many years. The suitability of microorganisms in this regard is determined by their ability to biosynthesize lipids with preferred fatty acid profile along with the concurrent utilization of energy-rich industrial waste. In this study, we isolated, characterized, and identified kefir yeast strains using molecular biology techniques. The yeast isolates identified were* Candida inconspicua*,* Debaryomyces hansenii*,* Kluyveromyces marxianus*,* Kazachstania unispora*, and* Zygotorulaspora florentina*. We showed that deproteinated potato wastewater, a starch processing industry waste, supplemented with various carbon sources, including lactose and glycerol, is a suitable medium for the growth of yeast, which allows an accumulation of over 20% of lipid substances in its cells. Fatty acid composition primarily depended on the yeast strain and the carbon source used, and, based on our results, most of the strains met the criteria required for the production of biodiesel. In particular, this concerns a significant share of saturated fatty acids, such as C16:0 and C18:0, and unsaturated fatty acids, such as C18:1 and C18:2. The highest efficiency in lipid biosynthesis exceeded 6.3 g L^−1^.* Kazachstania unispora* was able to accumulate the high amount of palmitoleic acid.

## 1. Introduction

Oleaginous yeasts are capable of accumulating over 20% of their cell mass as intracellular lipids and the production of microbiological lipids is defined as SCO (single cell oil) [1]. Oleaginous yeasts primarily belong to the genera* Rhodotorula*,* Rhodosporidium*,* Cryptococcus*,* Yarrowia*,* Trichosporon, *and* Lipomyces* [2–6]. The further exploration of the biological resources of the earth and food environment in this respect is still justified by the need to find new sources of biodiesel or nutritionally valuable fatty acids. Compared to other oleaginous microorganisms, such as filamentous fungi and algae, yeasts have a higher growth rate resulting in higher cell density [7]. Lipids produced by yeasts have similar fatty acid profiles compared to those of vegetable oils and are therefore considered as an alternative strategy for the production of second-generation fuels, including biodiesel [2, 8, 9]. The high yeast oils concentration and productivity can only be attained if a high yeast cell density is achieved at high productivity during the growth phase. High yeast cell density can only be achieved in well-selected bioreactors. As Koutinas et al. [10] concluded that the high costs represent also the capital and electricity consumption associated with the operation of classical field erected fermenters that are necessary in order to produce the microbial biomass and oil under fed-batch conditions. The engineering sciences have to solve these problems and deliver new solutions. However, also the high cost of culture media makes microbial oils less economically viable [11]. In recent years, it has become evident that biodiesel production should depend on agroindustrial wastes and renewable resources that do not compete with food and feed production. Several low-cost materials have been extensively studied as competitive alternatives for SCO production to make the production of yeast oil become economically viable. It is noteworthy that selection of oleaginous yeast should be directed toward the search for strains that can grow on cheap and available media, for example, the waste generated by the agrifood industry.

In developed countries, the problem of waste utilization, which is one of the most important priorities, needs to be addressed. Starch processing plants are arduous manufacturers of sewage. The production of potato starch involves formation of three types of organic wastes: potato juice, wastewater, and pulp. Potato wastewater is 10-fold diluted potato juice created during starch milk refining. To reduce the burden on potato wastewater, the starch processing plants perform the process of deproteinization by applying thermal-acid coagulation to remove proteins, which is then used in the production of animal feed [12]. Deproteinization step does not free potato wastewater from excess organic compounds [13], which still significantly burdens the wastewater.

Deproteinated potato wastewater (DPW) can act as a growth medium for yeast [14]. It contains raw protein, which includes soluble proteins and peptides, amino acids, minerals, and nitrogenous compounds [15]. The total organic carbon content in DPW is about 14-15 g L^−1^ [13, 16], and the concentration of reducing sugars, primarily represented by glucose, rhamnose, and galactose, fluctuates in the range of about 0.5–1%. To achieve a high yield in biomass, DPW must be supplemented with other available carbon sources, which contributes to both biomass yield and the content of intracellular components. In addition to saccharides, glycerol can be utilized as a carbon source in the yeast culture medium. The production of biodiesel involves the formation of crude glycerol, which has been studied for many years [8, 17]. At present, there is an urgent need to tackle the problem of utilization of glycerol because of its increasing annual production rate. As postulated by Almeida et al. [18], it has become a “waste-stream” instead of a valuable “coproduct.”

An inconvenient waste of the food industry is whey, which is generated in large quantities by the dairy industry. The possibility of biotechnological management of whey is largely dependent on the abilities of its microorganisms in assimilating or fermenting milk sugar—lactose. Kefir may be the natural environment for the yeast that have the ability to assimilate lactose. Therefore, it can be a source of lactose-positive yeast strains.

Kefir is a fermented milk product manufactured from milk subjected to lactic acid-alcohol fermentation in the presence of kefir grains. Kefir grains have a characteristic microflora that includes lactic acid-fermenting bacteria, acetic acid bacteria, and yeasts [23]. Kefir grains are clusters of symbiotic microorganisms held together by a polysaccharide matrix called kefiran [21], which may constitute up to 25% dry matter of kefir grains. Kefiran, formed by* Lactobacillus kefiranofaciens*, is a branched chain polysaccharide that is well soluble in hot water [28–31]. Kefir grains, also called kefir mushrooms, resemble cauliflower florets in their shape and reach up to a size of about 1–3 cm in diameter; they are not soluble in water and in most of the solvents. The sensory characteristics show that kefir is white or slightly yellow in color and have elastic consistency with compact structure [19, 20, 32]. According to the recommendations of Food and Agriculture Organization (FAO) and World Health Organization (WHO), 1 g of kefir should contain a minimum of 10^4^ colony forming units (cfu) of yeasts (FAO/WHO Food Standards: CODEX STAN 243-2003). Yeast microflora constituting 10–17% of kefir grains is predominantly composed of nonfermenting lactose yeasts (60–100% of total yeasts number) [23]. This makes them dependent on lactic acid bacteria, which use lactose as the carbon source. The pH decreases with the progress of lactic acid fermentation (lactic acid is produced), which favors the development of yeast. Yeast, in turn, stimulates the growth of lactic acid bacteria by the production of B-group vitamins and hydrolyze milk proteins by secreting proteolytic enzymes [20, 33]. Despite numerous reports concerning the nature of symbiotic systems in kefir grains, little is known about the formation of those yeasts and their biotechnological potential including their ability to accumulate intracellular fat.

The aim of this investigation was to isolate yeast strains from kefir and to study their biochemical behavior emphasizing their ability to accumulate lipids. The newly isolated strains were identified according to their internal transcribed spacer (ITS) sequence similarities. The studies regarding lipid accumulation abilities and fatty acid composition were performed. The concept of the utilization of potato wastewater as a source of assimilable nitrogen and other essential elements for oleaginous kefir yeast breeding was examined.

## 2. Material and Methods

### 2.1. Isolation Procedure

Natural kefirs were purchased from Polish manufacturers. All products were examined within their shelf life period. Kefir sample (10 g) was aseptically withdrawn and suspended in 90 mL of physiological saline. One mL culture from this dilution was loaded on Sabouraud dextrose agar with chloramphenicol and incubated at 28°C for 72 h. After incubation, the culture was evaluated for bacterial growth, and yeast colonies from the agar plate were transferred using streaking technique on Sabouraud dextrose agar and incubated at 28°C again for 72 h. It was then inoculated by streaking on YPD medium (composed of glucose 20 g L^−1^, peptone 20 g L^−1^, yeast extract 10 g L^−1^, and agar 20 g L^−1^, pH 5.5). The so-obtained strains were stored on YPD agar medium with pH 5.6 at 6°C, restreaking again on fresh medium every 3-4 weeks.

### 2.2. Identification Procedure

#### 2.2.1. Identification by Phenotypic Characteristic of Isolates

The yeast strains were identified using Api 20 AUX tests [BioMerieux, France] according to the manufacturer's instructions. Prior to testing, the isolates were subcultured on to Sabouraud dextrose agar and were incubated at 28°C in an aerobic atmosphere for 24–48 h.

#### 2.2.2. Identification by DNA Restrictions Analysis Method: Molecular Identification DNA Isolation

After 24 h culture in YPD medium, yeast biomass (2 cm^3^) was centrifuged at 716 ×g and 4°C for 10 min. Then, it was rinsed twice with sterile deionized water and suspended in 300 *μ*L lysis buffer (1 mM EDTA; 10 mM Tris-HCl, pH 8.0; 100 mM NaCl; 2% Triton X-100; 1% SDS) and then gently vortexed. The precipitate was incubated at a temperature of 37°C for 1 h. Then, 200 *μ*L of TE buffer (10 mM Tris-HCl, pH 8.0; 1 mM EDTA) was added. Then, 200 *μ*L of mixture containing phenol : chloroform : isoamyl alcohol at a ratio of 25 : 24 : 1 (pH 8.0) was added and vortexed for 60 s. The samples were centrifuged at 4472 ×g at 4°C for 10 min, and the upper phase was transferred to new sterile Eppendorf tubes. To this, 1 mL of 96% ethanol was added to obtain a precipitate. This precipitate was centrifuged at 8765 ×g for 10 min at 4°C and was washed with ice-cold 70% ethanol, dried, and suspended in 50 *μ*L sterile distilled water [34].

#### 2.2.3. DNA Amplification

DNA was amplified according to a previously described procedure [35]. The following primers were used in this experiment: ITS1 (5′ CGG GAT CCG TAG GTG AAC CTG CGG 3′) and ITS4 (5′ CGG GAT CCT CCG CTT ATT GAT ATG C 3′) each at a concentration of 20 pmol, 1.5 mM L^−1^ of MgCl_2_, 0.25 mmol L^−1^ of dNTP, and 0.5 U of Taq polymerase (Fermentas, Lithuania) were added to template DNA with a concentration of 300 ng *μ*L^−1^ (5 *μ*L). Amplification reaction was performed in a Mastercycler gradient thermocycler (Eppendorf company) using a program with the following parameters: temperature 94°C—5 min and then 34 cycles, 94°C—45 s; 60°C—1 min; 72°C—2 min; and 72°C—5 min. PCR reaction products were separated in 2% (w/v) agarose gel (Sigma) in 0.5x TAE buffer (60 min, 9.5 V/cm gel). Molecular weight markers of 1500–100 base pairs (bp) DNA (Thermo Scientific) were used to determine the molecular weight of the unknown bands obtained.

#### 2.2.4. DNA Digestion Using Restriction Enzymes

Two restriction enzymes, namely,* Hae*III (Fermentas, Lithuania) and* Rsa*I (Thermo Scientific), were used to digest the amplified DNA fragments as per the manufacturer's protocol. The digestion products were separated using electrophoresis on a 2% agarose gel (Sigma) in 0.5x TAE buffer and analyzed under UV light.

#### 2.2.5. Phylogenetic Analysis

Amplicons were sequenced using Genomed, Warsaw, Poland. DNA sequences were analyzed with basic local alignment search tool (BLAST). Phylogenetic analysis was performed using MEGA 7 by applying neighbor-joining algorithm [36].

### 2.3. Media and Growth Conditions

#### 2.3.1. Culture Media

The yeast strains were cultured using the following control media: (a) YPGlc containing 20 g L^−1^ peptone, 10 g L^−1^ yeast extract, and 50 g L^−1^ glucose; (b) YPLac containing 20 g L^−1^ peptone, 10 g L^−1^ yeast extract, and 50 g L^−1^ lactose; and (c) YPGly containing 20 g L^−1^ peptone, 10 g L^−1^ yeast extract, and 50 g L^−1^ glycerol (pH 5.6). All media were sterilized in an autoclave for 20 min at 121°C.

Potato wastewater was obtained from the processing Line of PEPEES SA Company (Łomża, Poland) during the potato campaign in autumn 2015, after coagulating the proteins using thermal-acid coagulation technique. The wastewater was sterilized for further evaluation (121°C/0.1 MPa/20 min) (HiCLAVE HG-80 autoclave, HMC Europe), as we had detected the presence of aerobic spore bacilli, mold, and yeast spores. DPW was centrifuged to remove precipitates formed during sterilization (3200 ×g/20 min) (Eppendorf 5810 Centrifuge), which was followed by the addition of glucose (50 g L^−1^), lactose (50 g L^−1^), or glycerol (50 g L^−1^). The pH was adjusted to 5.6 using 0.1 M NaOH and the entire mixture was sterilized according to the procedure described above. The experimental media supplemented with glucose, lactose, and glycerol will be, respectively, referred to as DPWGlc, DPWLac, and DPWGly in future of this manuscript.

#### 2.3.2. Preparation of Inoculum

Fifty-milliliter liquid YPD medium was inoculated with the selected strain from the slant cultures in round flat-bottom flask. The cultures were grown at 28°C (MaxQ 4000, Barnstead) for 24 h on a reciprocating shaker with a frequency of 200 cycles/min. The inoculum obtained by this method constituted material for inoculation of microculture in Bioscreen C system and for proper culture experiments.

#### 2.3.3. Screening of the Growth with the Use of Bioscreen C System

Growth of strains was analyzed in the experimental media using Bioscreen C apparatus (Oy Growth Curves Ab Ltd., Finland). Briefly, 270 *μ*L of the medium (control and experimental) and 30 *μ*L of inoculum (about 1 × 10^5^ cfu cm^−3^) were introduced to one microwell on the Honeycomb plate. The cultures were grown at 28°C under constant shaking, and their optical density (OD) was recorded for every 15 min (*λ*_420–580 nm_). Suitable maximum growth rates (*μ*_max_) and generation time were determined after preparing the growth curves. The coefficient of specific growth rate (*μ*) in time (*t*) was calculated using the formula, *μ*(*t*) = (ln⁡OD_*f*_ − ln⁡OD_*i*_)/(*t*_*f*_ − *t*_*i*_), where OD_*f*_ is final OD in the log phase, OD_*i*_ is initial OD in the log phase, *t*_*f*_ is time of log-phase termination, and *t*_*i*_ is time of log-phase onset.

#### 2.3.4. Experimental Culture Conditions

All cultures were grown in 500 mL flasks. Media (90 mL) were inoculated with inoculum constituting 10% of the culture volume. The cultures were incubated for 96 h at 28°C on SM-30 Control (Buehler, Germany) shaker at a frequency of 200 cycles min^−1^. Each culture variant was grown in triplicate.

#### 2.3.5. Examinations of Cell Morphology

The examination of the shape and size of the cells was conducted using the microscope (Opta-Tech 300, Poland) and microscopic camera (Opta-Tech) integrated OptaWiew7 (V:2.1.) software. The length (longer dimension), width (shorter dimension), and surface of minimum of 100 cells were measured.

#### 2.3.6. Determination of OD

OD of culture was determined after centrifugation of 2 mL of culture (1610 ×g/5 min) (Eppendorf mini Spin plus). The supernatant was decanted, and the biomass precipitate was suspended in 2 mL of distilled water. OD was measured spectrophotometrically at a wavelength of 600 nm against distilled water as blank (UV – 1800 UV/VIS, RayLeigh Analytical Instrument).

#### 2.3.7. Determination of Biomass Yield

To determine the biomass yield after completion of incubation, postculture fluid was transferred to a dried and weighed extraction thimble and centrifuged (2000 ×g/20 min) (Eppendorf Centrifuge 5804R). Then, the supernatant was decanted and the precipitate containing the biomass was washed twice with sterile distilled water followed by drying until a constant weight was achieved at 105°C (SML 32/250 Zelmed drier, Poland). Biomass yield was expressed in terms of grams of dry yeast per liter of culture medium (g d.w. L^−1^ medium). The biomass concentration at the beginning of culture was about 0.4 g d.w. L^−1^ medium for each strain.

#### 2.3.8. Lipid Analysis

After culturing, yeast biomass was centrifuged (2000 ×g/4°C/10 min) (Eppendorf Centrifuge 5804R). Then, it was rinsed twice with sterile deionized water and then dried at 80°C until a constant weight was achieved. The biomass yield was expressed in terms of grams dry yeast per liter of the culture medium (g d.w. L^−1^ medium). The lipid content in yeast biomass was determined according to the method of Bligh and Dyer and modified based on Zhang et al. [37], which involves lipid extraction using chloroform and methanol. Lipid content was expressed as % of cell dry weight (% CDW).

*(1) Relative Composition of Fatty Acids*. Fatty acid methyl esters were determined using the gas chromatographic technique coupled with flame ionization detector (GC-FID TRACE 1300, Thermo Scientific); methylation of fatty acids to methyl esters was performed prior to GC-FID analysis. Lipids were extracted using chloroform/methanol mixture and then the lipids were dissolved in hexane. Then, methylation of the lipid phase dissolved in hexane was performed using potassium methoxide (37°C, 12 h^−1^). The hexane layer was collected and subjected to chromatographic separation using Rtx-2330 column (60 m × 0.25 mm × 0.2 *μ*m). The samples were injected at 250°C (split 1 : 10). The following gradient of oven temperatures was applied: 50°C (3 min) and 3°C min^−1^ temperature increase to 250°C (5 min). The separation was conducted with a steady flow of carrier gas through the capillary column (1.6 cm^3^ min^−1^). The temperature of FID detector was set at 260°C. The methyl esters were identified based on the retention times of standards present in the mixture (GLC 461 Nu-Chek Prep. Inc. USA).

### 2.4. The Deproteinated Potato Wastewater Characterization

#### 2.4.1. The Selected Elemental Content

The dry matter content in DPW was determined by gravimetric method (105°C, 24 h, SML 32/250, Zetmet). The nitrogen content was determined by the Kjeldahl method (Büchi Digestion Unit K-435, Büchi Distillation Unit K-355) [16]. The elements that were estimated in DPW were P, K, Na, Ca, Mg, and Mn using inductively coupled plasma atomic emission spectroscopy (ICP-AES) (Thermo iCAP 6500). For this, the samples of deproteinated potato wastewater (2 mL) were mineralized (Buchi Digestion Unit K-435) using a mixture of nitric acid (5 mL) and perchloric acid (2 mL). After mineralization, the digested mixture was transferred by purging hydrochloric acid to 25 mL flasks and then filling them with deionized water. The wavelength for individual element was Ca: 315.8, 373.6, and 422.6 nm; K: 766.4 and 769.8; Mg: 279.5, 280.2, 285.2, and 382.9; Na: 588.9 and 589.5; and P: 177.4, 178.2, and 213.6. The results were expressed in terms of grams of element adjusted to a one liter of the culture medium. The measurements of the selected elements were also performed for synthetic YPGlc medium.

#### 2.4.2. Electrophoretic Analysis

Proteins were separated on a polyacrylamide gel electrophoresis under denaturing conditions (SDS-PAGE) using 3% thickening silica gel and 12% separating gel. Protein was denatured in a temperature range of 90–100°C for 5 min with concurrent shaking (Eppendorf Thermomixer Comfort, Germany). Then, electrophoresis was performed using a Mini Protean® 3 Bio-Rad device at constant current of 20 mA (voltage is 200 V) in 1x Tris-glycine buffer with a pH of 8.3. An appropriate molecular weight marker (Precision Plus Protein Dual Xtra Standards, Bio-Rad) was used to determine the mass of protein fractions. For visualization, the gel was stained with Coomassie Brilliant Blue R-250. Electrophoretically separated proteins were documented using a gel recording system (GelDoc 2000, Bio-Rad, France). Proteins were analyzed using the Quantity One version 4.2.1 (France) computer program [38].

### 2.5. Statistical Analysis of the Results

All values are mean of three separate experiments. The results obtained were statistically analyzed using the STATISTICA V.10 program (StatSoft Polska Sp. z o.o., Krakow, Poland). Analysis of variance (ANOVA) was performed using Tukey's test at *α* = 0.05 level of significance.

### 2.6. Calculation of Biodiesel Properties

The properties of fatty acids methyl-esters, namely, unsaturation degree (UN), cetane number (CN), length of chain (LC), low caloric value (LCV), flash point (FP), and viscosity (*μ*) were estimated based on the following equations:

(1) $$\begin{array}{c} UN=\frac{\left(1{\%}_{MU}+2{\%}_{DU}+3{\%}_{TU}\right)}{100} \end{array}$$

(see [39]),
  (2) $$\begin{array}{c} CN=\Sigma X_{ME}{CN}_{ME} \end{array}$$
(see [40]),
  (3) LC=RnCncn,LCV=29385,4+486,866LC−387,766UD kJ kg−1,μ=−1,8327+0,209794LC+0,738911UD+0,0166791LC2−0,16336LCUD+0,335547UD2 mm2 s−1
(see [39]),

where

 
*n*C_*n*_ is the number of carbon atoms of each fatty acids,
 
*c*_*n*_ is the percentage of weight of each methyl ester containing this fatty acids,
 
%_MU_ is the percentage of weight of monounsaturated methyl esters,
 
%_DU_ is the percentage of weight of di-unsaturated methyl esters,
 
%_TU_ is percentage of weight of tri-unsaturated methyl esters,
 
*X*_ME_ is the weight percentage of each methyl ester,
 
CN_ME_ is the cetane number of individual methyl ester.

## 3. Results

### 3.1. Identification of Kefir Yeast Isolates

**Figure 1:**
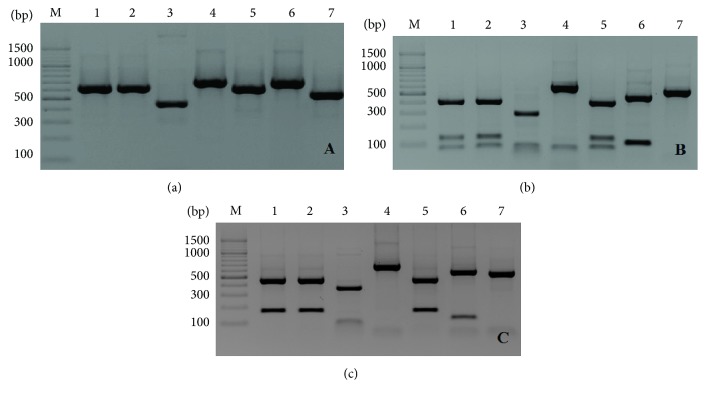
(a) Electrophoretic separation of PCR amplification products on 2% agarose gel (M—molecular marker; 1—*Debaryomyces hansenii *1; 2—*Debaryomyces hansenii *IG II; 3—*Candida inconspicua *IG 11; 4—*Kluyveromyces marxianus *IG 16; 5—*Debaryomyces hansenii *IG 01; 6—*Kazachstania unispora *IG 16; 7—*Zygotorulaspora florentina *IG 12). (b) Electrophoretic separation of yeast DNA digested with restriction enzyme* Hae*III (M—molecular marker; 1—*Debaryomyces hansenii *1; 2—*Debaryomyces hansenii *IG II; 3—*Candida inconspicua *IG 11; 4-*Kluyveromyces marxianus *IG 16; 5—*Debaryomyces hansenii *IG 01; 6—*Kazachstania unispora *IG 16; 7—*Zygotorulaspora florentina *IG 12). (c) Electrophoretic separation of yeast DNA digested with restriction enzyme* Rsa*I (M—molecular marker; 1—*Debaryomyces hansenii *1; 2—*Debaryomyces hansenii *IG II; 3—*Candida inconspicua *IG 11; 4—*Kluyveromyces marxianus *IG 16; 5—*Debaryomyces hansenii *IG 01; 6—*Kazachstania unispora *IG 16; 7—* Zygotorulaspora florentina *IG 12).

In this study, the degree of identification of yeast isolates based on API 20 AUX system was high, which was found to be more than 99%. This allowed the identification of seven strains from the isolates. The species identified were as follows:* Candida guilliermondii *1,* Candida guilliermondii *2,* Candida famata*,* Candida kefyr*,* Candida inconspicua*,* Saccharomyces cerevisiae*, and* Cryptococcus albidus *(Table 1). Limited identification opportunities of API 20 AUX system prompted us to perform the identification based on molecular tools. PCR analysis of the reaction products using ITS1 and ITS4 primers and restrictive cleavage using enzymes* Hae*III and* Rsa*I, as well as sequencing, were the basis for their identification (Table 1). Application of ITS1 and ITS4 primers to amplify DNA fragments of kefir yeast isolates causes an intensification of PCR products of about 460–700 bp (Figure 1(a)). The applied primers ITS1 and ITS4 caused strengthening of the amplification product by a value of about 640 bp in case of three isolates of* Debaryomyces hansenii*. In case of* C. inconspicua *IG 11,* Kluyveromyces marxianus *IG 1, and* Zygotorulaspora florentina* IG 12, the strengthening of the product after DNA fragment amplification was 460, 700, and 600 bp, respectively (Table 2).

We found, after electrophoretic separation of the PCR products as a result of obtaining of determined length of amplicons and the use of restriction enzyme H*ae*III, that the molecular weights of the PCR products for all strains of* D. hansenii *were 410, 140, and 90 bp, respectively (Figure 1(b)). Different length restriction fragments of* C. inconspicua *IG 11 (290, 90, and 80 bp),* K. marxianus *IG 1 (620 and 80 bp), and* Kazachstania unispora *IG 16 (550 and 130 bp) were obtained as a result of DNA digestion of the amplification products using restriction enzyme* Hae*III. In case of three isolates of* D. hansenii*, two bands with 450 and 190 bp were obtained as a result of digestion using* Rsa*I enzyme (Figure 1(c)). However, in case of* Z. florentina *IG 12, DNA digestion using* Hae*III restriction enzyme did not result in any new fragment, whereas digestion using* Rsa*I enzyme resulted in two fragments with 520 and 80 bp (Table 2). Table 1 shows the partial and complete gene sequences for 18S rRNA ITS1, 5.8S rRNA, ITS2, and 28S rRNA that were deposited and analyzed in GenBank, including their accession number. Figure S1, in Supplementary Material available online at https://doi.org/10.1155/2017/6061042, presents the differences between the yeast isolates using the phylogenetic tree [41].

### 3.2. Morphology and Cell Dimensions of Kefir Yeast Isolates

All isolated yeast strains formed colonies of white to various shadows of cream and beige color on the solid medium and were mostly characterized by shape specific for yeasts. Strains* K. marxianus *IG 1*, Z. florentina *IG 12*, K. unispora *IG 16, and three strains of* D. hansenii *formed glossy colonies. In liquid media, most of the examined strains were growing in the whole medium volume (diffusion growth) during stationary culture. A ring on liquid medium surface was formed by* D. hansenii *1 and* C. inconspicua *IG 11 strains.

The lowest dimensions in YPD medium (Table S1) were characteristic for the cells of both* D. hansenii *IG II strains. Cells, longer than 3 *μ*m, belonged to* C. inconspicua *IG 11 strain. The cell width of a particular strain was from 1.59 to 2.58 *μ*m, and it is noteworthy that, in this case, the dispersion of the results was much lower than in the case of the length. The cell morphology closest to sphere was determined by definition of shape coefficient expressed as a ratio of cell length to its width. The value of shape coefficient for such cells should amount to 1. The strains whose cells were of spherical shape included all* D. hansenii* strains and* Z. florentina *IG 12. The shape coefficient between 1 and 2 was for the following strains* K. unispora *IG 16,* K. marxianus *IG 1, and* C. inconspicua *IG 11. Their cell morphology can be described as oval or ellipsoidal.

### 3.3. Characteristics of DPW

The physicochemical parameters of wastewater obtained from the process line after deproteinization stage shows Table 3. The dry matter content in 100 cm^3^ exceeds 3.3 g, and the amount of reducing sugars was found to be 0.4 g. An average nitrogen level was found to be 0.16 g 100 cm^−3^, and the protein content calculated based on nitrogen level was found to be 1 g 100 cm^−3^. The wastewater contained large amounts of potassium (over 0.4%). The electrophoretic analysis of wastewater did not confirm the presence of proteins of a weight lower than 250 kDa (Figure S2).

### 3.4. Growth of Kefir Yeast in the Examined Media

Isolated yeast strains were able to assimilate the examined carbon sources. The cells grew the fastest in the glucose-supplemented media with peptone and yeast extract. The use of lactose and glycerol significantly reduced the specific growth rate (Table S2). We observed that the growth of the yeast isolates in media with wastewater was characterized by an extended lag-phase (data not shown) and lower specific growth rate compared to the control YP medium. Supplementation of lactose and glycerol in these media reduced the specific growth rate compared to glucose. Only in case of* D. hansenii* 1 and* D. hansenii* IG 01, glycerol in the medium increased the proliferation rate of the cells.

The highest biomass yield (in the range of 13.5–14.8 g L^−1^) was observed in the YPGlc medium in case of three strains of* Debaryomyces *genus and* Z. florentina* IG 12 (Table 4). The lowest biomass yield (11 g L^−1^) in the control media (YP) with glucose and lactose was noted for* K. unispora* IG 16 culture. The differences in fertility of strains depending on the tested carbon source with wastewater were much smaller, for example, in the media with lactose, average yields of examined strains' biomass ranged from 10.9 to 12.4 g L^−1^ and in case of glycerol from 10.3 to 11 g L^−1^. Biomass productivity did not reach the value of 0.2 g L^−1^ h^−1^ in any tested medium variant, both the control and the wastewater.

### 3.5. Intracellular Lipid Content

**Figure 2:**
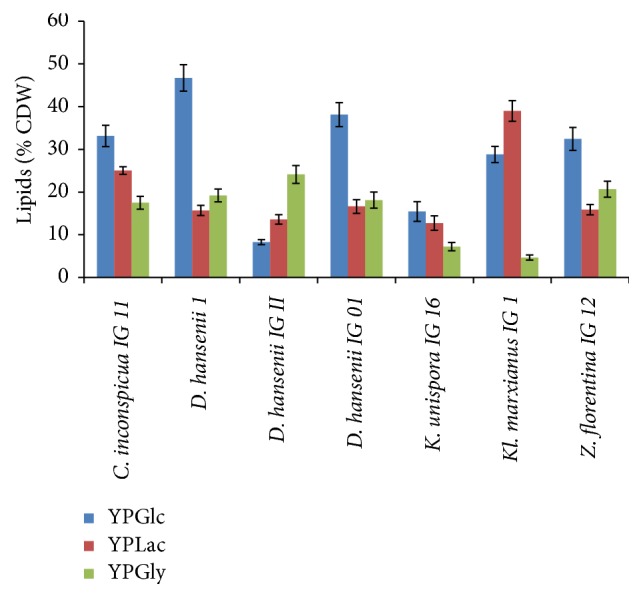
Total lipid production (% cell dry waste (CDW)) in kefir yeast biomass after culturing in YP media supplemented with glucose, lactose, and glycerol.

The highest lipid content after incubation in YP media with glucose was found in the biomass of* D. hansenii* 1 strain (CDW more than 46% after 96 h of culturing) (Figure 2). Fat content greater than 20%, which is a feature of oleaginous microorganisms, was found in case of* D. hansenii* IG 01,* C. inconspicua *IG 11,* Z. florentina* IG 12, and* K. marxianus *IG 1 strains. Lower fat content was found in the same medium with lactose supplementation. The exception was* K. marxianus* strain, which accumulated 39% of fats in dry weight. Glycerol, compared to glucose, in the medium with peptone and yeast extract, generally reduced lipid content in the group of examined strains. An exception was* D. hansenii* IG II strain, whose biomass after culturing in YPGly medium was characterized by lipid content more than 24%. The volumetric yield of lipid biosynthesis in control media was the highest in the case of* D. hansenii* 1, which was 6.31 g L^−1^, for* D. hansenii* IG 01 it was 5.65 g L^−1^, and for* Z. florentina* IG 12 it was 4.44 g L^−1^. In the media supplemented with glycerol, the highest volumetric yield was determined for* D. hansenii* IG II (3.28 g L^−1^) and, concurrently, it was the highest compared to other carbon sources (Table 4). Lactose profitably increased the ability of cells to biosynthesize lipids in case of* K. marxianus* IG 1 and* D. hansenii* IG II strains.

**Figure 3:**
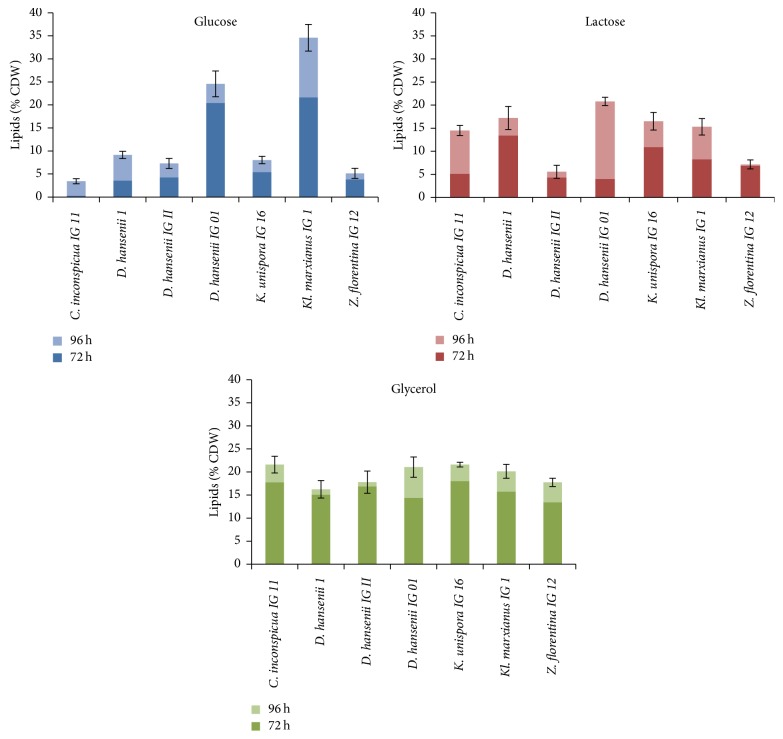
Effect of culture time on lipid content, expressed as % cell dry waste (CDW): dark-colored bars cell dry waste—72 h and light-colored bars—96 h, in deproteinated potato wastewater (DPW) media with glucose, lactose, and glycerol.

Lipid content in dry biomass after the culturing in DPWGly medium exceeded 20% for* C. inconspicua*,* D. hansenii* IG 01,* K. unispora* IG16, and* K. marxianus* IG I strains (Figure 3). In the latter two strains, it was higher than the content in biomass after culturing in YP medium. Compared to the other carbon source, glycerol profitably affected the yield of lipids synthesis in the biomass of* K. unispora* IG 16,* D. hansenii* IGII,* C. inconspicua* IG 11, and* Z. florentina* IG 12 obtained after culturing in medium with the wastewater. Similar pattern was found for the volumetric (*Q*_*L*_) yield of lipids (Table 4). The lactose in DPW medium, compared to YP medium, significantly increased lipid content in the biomass of* D. hansenii* IG 01 and* K. unispora*. Wastewater as a source of nitrogen significantly reduced lipid content in the biomass of* C. inconspicua*,* D. hansenii* IG II,* K. marxianus*, and* Z. florentina*.

The use of DPW medium and glucose as carbon source revealed major differences in the ability of the examined strains for lipids biosynthesis. The share of this component higher than 20% g^−1^ d.w. biomass was only noted for* D. hansenii* IG 01 and* K. marxianus* IG I (Figure 3). Lipid content in the biomass of other strains was lower and did not exceed 10%. The highest volumetric yield was noted for* K. marxianus* (3.56 g L^−1^) and* D. hansenii* IG 01 strains (2.80 g L^−1^). It should be noted that the values were significantly lower than those achieved for the strains in media with yeast extract and peptone. Thus, the comparison of the volumetric yield of fats biosynthesis in control YP media with glucose and in experimental media with wastewater is favorable for the control medium.

Based on these findings, we can conclude that lipid content in d.w. of the examined yeast biomass was dependent on the duration of the culture, and, in all variants of the examined media, the share of this intracellular component increased after 72 h (Figure 3).

### 3.6. pH Changes

The initial pH of all media was 5.6. Cultures of examined yeast strains in all variants of media, experimental and control, caused significant changes in pH (Table 4). During the culture of yeast strains, the pH value of YP medium supplemented with glucose was found between 5.1 (*C. inconspicua*) and 7.31 (*K. marxianus *IG 1). In the YPLac, pH was in the range of 5.17–7.69, and, using glycerol, it was in the range of 5.04–7.63. Alkalization during culturing was observed for all strains in the experimental DPW media. The highest pH values (8.01–8.63) after 96 h were noted for the medium with wastewater supplemented with glycerol.

### 3.7. Fatty Acids Composition

**Figure 4:**
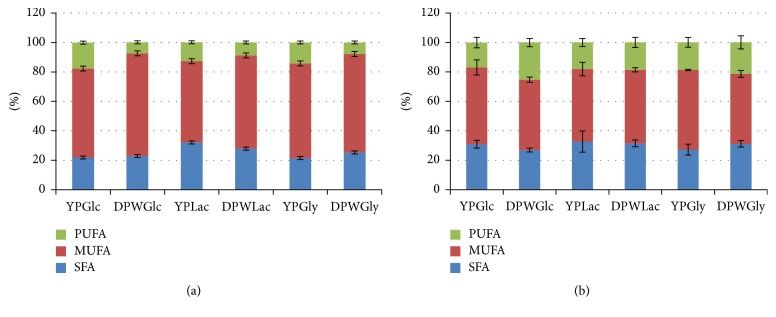
Total content of particular groups of fatty acids after culturing in all media supplemented with glucose, lactose, and glycerol on an example of* Kazachstania unispora* (a) and* Debaryomyces hansenii* (average values for all isolates) (b). SFA: saturated fatty acid; MUFA: monounsaturated; PUFA: polyunsaturated fatty acids; YPGlc: yeast peptone agar supplemented with glucose; YPLac: yeast peptone agar supplemented with lactose; YPGly: yeast peptone agar supplemented with glycerol; DPWGlc: deproteinated potato wastewater supplemented with glucose; DPWLac: deproteinated potato wastewater supplemented with lactose; DPWGly: deproteinated potato wastewater supplemented with glycerol.* Note*. Tukey's test, *α* = 0.05.

The results of fatty acid profile after culturing yeast isolates in control (YPD) media supplemented with glucose are presented in Table 5. All examined yeast isolates demonstrated a large share of C18 fatty acids, among which oleic acid (C18:1* cis-9*) was predominant. Its greatest share (more than 60%) was found in lipids extracted from* D. hansenii* IG 01 biomass. A small amount of C18 fatty acids was found in the lipids of* K. unispora* strain; there was no C18:2 (linoleic acid) detected. In addition, this strain was characterized by the largest share of palmitoleic acid (C16:1) (more than 39% for the YPGlc medium) and arachidonic acid (C20:4 n-6) and showed the presence of dihomo-gamma-linolenic acid (DGLA; C20:3 n-6). Docosadienoic acid (C22:2) was only found in case of two strains, that is,* K. unispora* and* Z. florentina*. Alpha linolenic acid (ALA, C18:3 n-3) was detected in the biomass of almost all strains. The largest share of polyunsaturated fatty acids (PUFAs) for most examined strains was detected in the control media supplemented with glucose (Figure 4). YP media supplemented with glycerol, compared to glucose and lactose, resulted in an increase in the total monounsaturated fatty acids (MUFAs), which can be correlated with a decrease in the pool of saturated fatty acids (SFAs). An exception was* D. hansenii* IG 01 strain, because a significant increase in the total content of PUFA was noted during its culture in medium supplemented with glycerol. However, an effect of glycerol on total MUFA content (mg L^−1^) was no longer so evident. The lowest amount of C18:1 (less than 20%) among all strains was determined in fat extracted from* K. unispora* biomass obtained after culturing in DPWGlc, similar to YPGlc medium. Among other examined strains, the amount of oleic acid was significant and accounted for more than 45% of the total fatty acid content in the biomass of* C. inconspicua*,* K. marxianus*, and three strains of* D. hansenii*.* K. unispora* strain, regardless of carbon source, was characterized by the largest share of palmitoleic acid and the lowest share of PUFA.

The largest share of PUFA after culturing in experimental media with wastewater and glucose was found in the biomass of the three strains belonging to* D. hansenii* species (from 19.3 to 30.6% CDW). Regardless of the used carbon source, the culture of two isolates,* C. inconspicua* and* K. unispora,* in DPW media resulted in fat containing a significantly higher MUFA contribution. Such dependency has not been found for other strains (Figure 4).

## 4. Discussion

The molecular studies aimed at identification of general yeast species have emphasized either coding, that is, D1/D2 variable domains of a large subunit rDNA, or noncoding, that is, ITS regions of the DNA [42]. Databases of ITS sequences were available for molecular classification and identification of yeasts. Coding regions of the 18S, 5.8S, and 28S nuclear rRNA gene evolve slowly, are relatively conserved among fungi, and provide a molecular basis of establishing phylogenetic relationships [43, 44]. For the three isolates of* D. hansenii*, the molecular weights of amplification products were similar to those published by Fujita et al. [45] and Połomska et al. [46]. Our results agree with the results published by Fujita et al. [45], after an application of the same primers for* C. inconspicua* (weight of about 449 bp). Under the same conditions, in case of* K. unispora*, Santiago-Urbina et al. [47] obtained amplification product with a molecular weight of 790 bp, whereas, in this study, the same strain showed a molecular weight of 690 bp. We observed that, after electrophoretic separation of the PCR products as a result of a determined length of amplicons and the use of restriction enzyme H*ae*III, the molecular weights of the PCR products for all strains of* D. hansenii* yeast were similar to those presented by Esteve-Zarzoso et al. [48]. The authors using the same primers for* D. hansenii* 10386 strain obtained PCR products with the molecular weights of 420, 150, and 90 bp. According to the data presented by Esteve-Zarzoso et al. [48], as a result of DNA cleavage with* Hae*III enzyme, the yeast of* K. marxianus* species demonstrated the presence of two bands with their molecular weights similar to those obtained in this study.

The wrong identification of* D. hansenii* (*C. famata* teleomorph) as* P. guilliermondii* (*C. guilliermondii* teleomorph) was pointed by Nishikawa et al., who also emphasized that these strains are extremely difficult to differentiate phenotypically [49, 50]. In [51], Desnos-Ollivier et al. analyzed the dozens of isolates of different origin with above synonyms and their aim was to assess, using nucleotide sequences, whether phenotypic identification was correct. They concluded, like others [52], that sequencing of ITS (or D1/D2 region) is a good tool for differentiating the species more frequently confused with* D. hansenii.*

Most of the identified strains in studies discussed herein were previously isolated from kefir or kefir grains by other authors (Table 6). The studies confirming the occurrence of* Z. florentina* yeast in kefir samples have not been published so far. But in 2003, Fröhlich-Wyder isolated* Zygosaccharomyces florentinus *strain from kefir [19]. It is noteworthy that Kurtzman [53] got a proposal of the new genera* Zygotorulaspora* from the perspective of the multigene sequence analysis. Until now, two species are accepted:* Zygotorulaspora florentinus* (Basionym:* Zygosaccharomyces florentinus*) and* Zygotorulaspora mrakii* (Basionym:* Zygosaccharomyces mrakii*).

All examined yeast strains were able to assimilate glucose, glycerol, and lactose. According to the CBS-KNAW Collections data, the* C. inconspicua* yeast does not have the ability to assimilate lactose. However, a study has demonstrated its ability to assimilate and ferment whey containing 5% lactose [54] along with a 1.9% v/v production of alcohol. The isolate of* C. inconspicua* IG 11 used in this study fermented glucose with a good yield and demonstrated a high resistance to ethanol (18%) [55]; however, it did not ferment lactose. Leite et al. [32] listed* Kazachstania unispora *as the nonlactose fermenting yeast. Our earlier analysis confirmed the lack of ability of this isolate to ferment lactose [55].

The study on wastewater composition confirmed that it can be a valuable source of nitrogen, potassium, phosphorus, and other elements in yeast cultures. High percentage of potassium especially in the wastewater results from the characteristics of the raw material—potatoes, which are a rich source of this element. However, it should be taken into account that the chemical composition of this waste depends on many factors, which include, inter alia, potato variety, type of soil and fertilization, and the technology and the efficiency of obtaining starch and deproteinization. Compared to the waste originating from a different region, the wastewater used in this study was characterized by threefold lower share of calcium and lower concentration of protein compounds [13]. Similarly, wastewater obtained in the laboratory deproteinized with sulfuric acid was characterized by a higher share of nitrogen [16].

The available nitrogen forms in wastewater are proteins, amino acids, purines, and pyrimidines. The easy soluble protein fractions get into a potato wastewater containing 2–5% of solids, of which crude protein represents about 35% [56, 57]. Potato proteins have been classified by Pots et al. [58] into three groups: patatin (43 kDa) comprises 38%, protease inhibitors (4.3–25.0 kDa) 50%, and other proteins up to 12% in potato wastewater. Potato carboxypeptidase inhibitor with a molecular weight of 4.3 kDa is extremely thermostable as though it remained soluble even when the high temperatures had been used [59]. There is a probability of the presence of thermostable protein fractions in wastewater after the separation of potato proteins intended for animal feed. However, the electrophoretic analysis of wastewater which we used did not confirm the presence of proteins of potato carboxypeptidase inhibitor fraction or any protein of a weight lower than 250 kDa (Figure S2). Our studies have confirmed the high effectiveness of the deproteinization process in the starch-producing company.

Intracellular lipid content was dependent on the strain and the applied medium. It is assumed that the process of fat accumulation is induced by a C : N ratio of greater than 20, and in the case of some microorganisms it may be higher than 70 [9]. For oleaginous* Lipomyces starkeyi *yeast, cultured in a synthetic medium containing glucose as the carbon source and ammonium sulfate as the nitrogen source, the highest lipid content in the biomass amounting to 68% CDW was obtained when the initial C : N ratio was 150 [60]. The effect of the C : N ratio on lipid metabolism has been investigated for a number of oleaginous yeasts and molds [8, 61–65]. The C : N : P ratio in the applied control media (YP) with glucose was 6.45 : 1 : 0.93, in media with lactose it was 6.78 : 1 : 0.93, and with glycerol it was 6.29 : 1 : 0.93. Compared to them, the media prepared based on wastewater were characterized by twofold higher C : N ratio. In the media with glucose C : N : P ratio was 12.3 : 1 : 0.204, with lactose it was 12.93 : 1 : 0.204, and for the media with glycerol it was 12.06 : 1 : 0.204. It should be noted that the examined media were not characterized by the recommended excess of carbon with respect to nitrogen, and despite this fact the share of lipids in dry weight of the selected strains was significant. Phosphorus is an element essential for the proper growth of the yeast, but its concentration does not affect in any significant manner the intracellular lipid biosynthesis by these microorganisms. It may, however, affect the fatty acid profile. For example, lipids extracted from* C. utilis *yeast biomass obtained from the medium with low phosphate content were characterized by a modified composition [66]. The process of synthesis and accumulation of intracellular lipid is also influenced by the presence of cations necessary for the proper activity of ATP-citrate lyase, a key enzyme of fatty acid synthesis de novo [67]. The activity of the enzyme is absolutely dependent on the presence of Mg^2+^. The magnesium ion content in potato wastewater was 0.0236 g 100 cm^−3^ and was fourfold higher than in YPD medium [16]. Higher supply of magnesium can be critical to the system. Moreover, Mn^2+^ and Co^2+^ could partially substitute Mg^2+^ deficiency. In this study, Co^2+^ content was not determined, but high concentration of Mn^2+^, that is, 0.181 mg 100 cm^−3^, was found in the wastewater.

Until now, pure glycerol being used as sole carbon source supported the growth and lipid accumulation of* Candida freyschussii *and many carotene-producing strains, for example,* Rhodotorula glutinis*,* R. glutinis *var*. rubescens*, and* R. mucilaginosa* [34, 68, 69]. Using a pure form of glycerol as a feedstock for SCO production would be cost-prohibitive for larger-scale production. The composition of the waste glycerol depends on many factors and contains methanol, mono- and diglyceride, free fatty acids, and soap [70]. These substrates may affect the growth and lipid biosynthesis by the yeast. However, no negative effect on growth was observed for* Rhodotorula *strain when metabolizing biodiesel-derived glycerol, even when using a higher concentration of waste glycerol [71]. During incubation with glycerol and deproteinated potato wastewater yeast isolates showed worse ability to biosynthesis lipids than other strains in media containing glycerol. For example, strain* R. glutinis* var.* rubescens* produced 4.73 g lipids L^−1^ [34] and* Yarrowia lipolytica* over 6 g L^−1^, and* Candida pulcherrima* 7,3 g L^−1^, and* Zygosaccharomyces rouxii* over 5 g L^−1^ [72]. On the other hand kefir isolates showed a better ability to synthesize lipids compared to, for example,* Candida guilliermondii *NRRL Y-2075 and several other species (less than 1 g L^−1^ when grown in the defined medium with biodiesel-derived glycerol) analyzed by Papanikolaou et al. [73].

Studies regarding the use of lactose as carbon source during the culture of oleaginous microorganisms are very scarce. To date, only one study related to the effect of lactose on the growth and accumulation of fats by fungi of* Trichosporon* genus has been conducted [74]. In addition, a study on use of whey for the synthesis SCO involving* Saprolegnia diclina* fungi that accumulated the lipids at the level of 14.05 g 100 g^−1^ has been performed [75].

Our results confirm the well-known theory of efficient lipid synthesis in the late stationary phase and at the same time suggest the need to optimize the culture time. We found a large increase in biomass yield and fat content between third and fourth day in the media with lactose, which suggests that lactose is assimilated in a slow manner compared to other carbon sources, which resulted in the biosynthesis of fats observed on fourth day.

The optimal pH value of the culture medium with respect to promoting lipid biosynthesis by the yeast is in the range of 4–8, depending on the type of microorganism and the used carbon source [76]. An increase in pH of DPW media, regardless to the strain, was observed during the cultures. The phenomenon of strong alkalization of the media prepared based on DPW was also observed by Nowak et al. during the culture of filamentous fungi [77]. In addition to the available organic nitrogen sources, DPW also contains its unavailable forms in the form of compounds such as amines or imines. Condensation reactions between carbonyl groups contained in the sugars and primary amino groups occur during the preparation of starch and then the separation of potato proteins. The resulting imines (so-called Schiff base) undergo spontaneous rearrangement to the so-called Amadori compounds, which, in turn, polymerize to a brown product, which is poorly characterized so far. Probably, the unavailable organic forms of nitrogen are formed during the culture in the medium and thus may be the reason for alkalization of the medium. However, it should be noted that nitrogen depletion from the medium induces lipids biosynthesis, which causes degradation of cAMP (cyclic adenosine monophosphate) to ammonia and ammonium ion, which may also be the cause of alkalization.

Yeast growth is more effective when the pH of the medium is slightly acidic, and, therefore, alkalization of the environment may be a stress factor for the cell. A strong alkalization of the environment during yeast culture in DPW disturbs the homeostasis of nutrients and affects an expression of genes of glucose uptake and metabolism. The high pH causes a temporary drop in the concentration of cAMP and inhibition of protein kinase activator (PKA, protein kinase A). Consequently, the transcription factor Msn2/4p is subject to a rapid activation in the nucleus and activates gene related to stress, related, inter alia, to trehalose synthesis [78]. Perhaps for these reasons, lower content of fat was noted during the culture in DPW medium with glucose compared to other carbon sources. The pH control in DPW media during the cultures aimed at increasing intracellular biosynthesis of fats appears to be an important issue to be explored. Providing yeast cells with optimum pH during the culture in media with deproteinized wastewater could result in higher biomass yield and more efficient biosynthesis of fats.

According to the data described in the literature, the temperature of yeast culture aimed at increasing fats biosynthesis should be 25–30°C. It affects the proliferation rate of the yeast cells and fatty acid composition [5]. Culturing yeast at lower temperatures results in an increased PUFA content. This was proved by Amaretti et al. [65] using* Rhodotorula glacialis *DBVPG 4785, during which the share of PUFA in biomass, after culturing at a temperature of 0°C, was the highest and amounted to over 55%.

Fats synthesized by oleaginous yeast primarily contain the following fatty acids: myristic (C14:0), palmitic (C16:0), stearic (C18:0), oleic (C18:1), linoleic (C18:2), and linolenic acid (C18:3) [2]. The primary fatty acid present in the yeast lipids is oleic acid, and it may be even more than 70% (w/w) (Papanikolaou et al., 2011). The first analysis of the lipids of* D. hansenii *was done by Merdinger and Devine [79]. Our results confirmed fatty acid composition for* D. hansenii* isolates [80]. The fatty acids composition presented remarkable changes as function of incubations time and throughout the life cycle of* Debaryomyces*. The levels of oleic and palmitoleic acids gradually increased during both the growth phase and the lipid accumulation phase, which coincides with ascosporogenesis, while the concentrations of linoleic, stearic, and palmitic acids decreased towards the start of ascospore formation by* D. etchellsii* [81]. Compared to* D. etchellsii*, kefir's isolates of* Debaryomyces* are characterized by significantly higher stearic acid contribution and lower palmitoleic and linoleic acids (in the same growth phase). Fatty acid composition of* Kluyveromyces marxianus* isolated strain is very similar to* Kluyveromyces fragilis* CBS 398 [82]. However, isolated strain is distinguished by a significantly lower proportion of linoleic acid.* Kazachstania unispora* was able to accumulate the high amount of palmitoleic acid. Palmitoleic acid-rich diets have also been reported to improve circulating lipid profile, resulting in reduced total and LDL cholesterol [83]. Palmitoleic acid has been shown to prevent *β*-cell apoptosis [84] and inhibit the growth of Gram-positive bacteria [85]. Two limited availability sources having high concentrations of palmitoleic acid are sea buckthorn and macadamia nut oil [86]. Regardless of the carbon source, the use of deproteinated potato wastewater promoted palmitoleic acid biosynthesis. The maximum contribution (60%) of this fatty acid in* Kazachstania unispora* fat was detected after incubation in DPWGlc medium. The suitability of a microbial strain for producing palmitoleic acid or another fatty acid is dictated by several factors, namely, a combination of a high yield of biomass, proportion of the fatty acid in total FA, and a high percent lipid content under the chosen culture conditions.

The possibility of using fat as a raw material in the production of biodiesel depends on the different physicochemical characteristics of the fuel. The transesterification of microbial oils with a methanol yields to the corresponding monoalkyl esters so the chemical structure of the fatty acids methyl esters influences fuel properties. The successful commercialization of vegetable oils as biodiesel sources has been accompanied by the development of international standards. Some biodiesel standards are European standard EN 14124 and American Society for Testing and Materials ASTM D6751. In our study biodiesel properties were determined using mathematical equations based on the fatty acids compositions. An industrially used biodiesel is primarily composed of palmitic, stearic, oleic, linoleic, and linolenic acids. Biofuels used in Europe are characterized by certain specification, according to which the content of linolenic ester acid should not exceed 12% and the methyl esters of polyunsaturated fatty acids (containing, inter alia, four double bonds)—1% [87]. In addition, a study has demonstrated that the fatty acid exhibiting the best properties affecting improved quality of biodiesel is oleic acid [88]. The presence of unsaturated bonds in the molecule is the primary cause of autoxidation of fats; thus the presence of excess of PUFA esters in the biodiesel considerably reduces its oxidative stability. Culturing yeast at lower temperatures results in an increased PUFA content what is one of the cold-adaptation strategy and unsaturated index for oils is higher. For example, UN for* K. marxianus* fat received in 4°C incubation was 1,06 and only 0,75 in 30°C [3]. In addition, the longer the carbon chain of the fatty acid, the higher the fuel viscosity, which unprofitably affects the structural components of cars [89].

The fatty acid composition characteristic for the examined kefir yeast isolates is therefore appropriate in relation to the European Union (EU) requirements for biofuels, and, therefore, they can be a source of SCO. They contain C16:0, C18:0, and C18:2 acids, in particular C18:1. Fats from all strains contained no more than 2.6% of linolenic acid (C18:3 n-3). The suitable selection of carbon source during culture can reduce the amount of PUFA with more than four double bonds. Biodiesel properties based on the fatty acids composition of the microbial oil producers by kefir yeast strain isolates in different media are shown in Table 7. Cetane number is a primary indicator of fuel quality which is related to the ignition delay time. Cetane (hexadecane, C_16_H_34_) is the high quality standard on the cetane scale with an assigned CN of 100. For diesel fuel higher CNs have been correlated with reduction nitrogen oxides (NO_x_) exhaust emission [88]. Unsaturated molecules have higher flame temperatures; thus they increase the formation of NO_x_ during combustion. According to EN 14214 and ASTM D6751 cetane number of biodiesel should have minimum value of 54 and 47, respectively. The cetane number of kefir yeast oil was high in almost all cases (Table 7). Also flash points are more than 122°C (according to EN 14214) making the kefir yeast oils safe to store. Low FP may indicate the presence of methanol in biodiesel [39]. The high kinematic viscosity could affect the atomization of fuel on injection and has impact on injection and combustion. The predicted viscosity of yeast oil produced by the isolates was in all cases high and it means that it would not meet European standards, only ASTM D6751. Factors such a double bond configuration influence viscosity while double bond position affects viscosity less [88]. Another important parameter of a fuel is the low calorific value, which represents the amount of heat transferred to the chamber during combustion and indicates the available energy in a fuel [90]. The average low calorific value of biodiesel all kefir yeast (37,5 MJ kg^−1^) is lower than petrol (46 MJ kg^−1^) and diesel fuel (43 MJ kg^−1^), but higher than coal (32–37 MJ kg^−1^) [91].

In conclusion, the isolated kefir yeast strains have demonstrated their ability to biosynthesize intracellular lipids with a concurrent utilization of deproteinated wastewater in this study. Regardless of the carbon source, the use of deproteinated potato wastewater promoted palmitoleic acid biosynthesis by* Kazachstania unispora*.

## Supplementary Material

Supplementary materials contain information about dimensions of the cells of kefir yeast strains and its maximum growth rates.

## Figures and Tables

**Table 1:** Results of an identification of kefir yeast isolates using API 20 AUX and genetic analysis.

API name isolate identification	Origin	bp	Name of isolate GenBank number
*Candida* *inconspicua*/ *Candida* *krusei* 99.5%	1 caaaaaacaa aactttcaac aacggatctc ttggttctcg catcgatgaa gagcgcagcg 61 aaatgcgata cctagtgtga attgcagcca tcgtgaatca tcgagttctt gaacgcacat 121 tgcgccctct ggtattccgg agggcatgcc tgtttgagcg tcgtttcctt cttgcttgcg 181 agcagaaatg ggggggccct ggcattgggg ccgctctgaa aagaaacgtt gcgggcgaag 241 cgaactatga gtaggacgct tggccgccga acttaataca taagctcgac ctcaaatcag 301 gtaggaatac ccgctgaact taagca	326	*Candida inconspicua *IG 11 LC164191.1

*Candida* *guilliermondii *1 99.1%	1 acttttgctt tggtctggac tagaaatagt ttgggccaga ggtttactga actaaacttc 61 aatatttata ttgaattgtt atttatttaa ttgtcaattt gttgattaaa ttcaaaaaat 121 cttcaaaact ttcaacaacg gatctcttgg ttctcgcatc gatgaagaac gcagcgaaat 181 gcgataagta atatgaattg cagattttcg tgaatcatcg aatctttgaa cgcacattgc 241 gccctctggt attccagagg gcatgcctgt ttgagcgtca tttctctctc aaaccttcgg 301 gtttggtatt gagtgatact cttagttgaa ctaggcgttt gcttgaaatg tattggcatg 361 agtggtactg gatagtgcta tatgactttc aatg	394	*Debaryomyces hansenii *1 LC126697.1

*Candida* *famata* 99.9%	1 gaacttttgc tttggtctgg actagaaata gtttgggcca gaggtttact gaactaaact 61 tcaatattta tattgaattg ttatttattt aattgtcaat ttgttgatta aattcaaaaa 121 atcttcaaaa ctttcaacaa cggatctctt ggttctcgca tcgatgaaga acgcagcgaa 181 atgcgataag taatatgaat tgcagatttt cgtgaatcat cgaatctttg aacgcacatt 241 gcgccctctg gtattccaga gggcatgcct gtttgagcgt catttctctc tcaaaccttc 301 gggtttggta ttgagtgata ctcttagttg aactaggcgt ttgcttgaaa tgtattggca 361 tgagtggtac tggatagtgc tatatgactt tcaatgtatt aggtttatcc aactcgttga 421 atagtttaat ggtatatttc tcg	443	*Debaryomyces hansenii *IG 01 LC164190.1

*Candida* *guilliermondii *2 99.1%	1 cttttgcttt ggtctggact agaaatagtt tgggccagag gtttactgaa ctaaacttca 61 atatttatat tgaattgtta tttatttaat tgtcaatttg ttgattaaat tcaaaaaatc 121 ttcaaaactt tcaacaacgg atctcttggt tctcgcatcg atgaagaacg cagcgaaatg 181 cgataagtaa tatgaattgc agattttcgt gaatcatcga atctttgaac gcacattgcg 241 ccctctggta ttccagaggg catgcctgtt tgagcgtcat ttctctctca aaccttcggg 301 tttggtattg agtgatactc ttagttgaac taggcgtttg cttgaaatgt attggcatga 361 gtggtactgg atagtgctat atgactttca atgtattagg tttatccaac tcgttgaata 421 gtttaa	426	*Debaryomyces hansenii *IG II LC164187.1

*Saccharomyces cerevisiae* 61.0%	1 gccgaaccag cgcttaattg cgcggtttgg tgggtctctg tagctcagta gcactattac 61 acactgtgga gattttataa ttctttgcat gcttctttgg gcagcttcgg cagcccagag 121 gtaacaaaca caaacaactt tgtaatattt ttaacccagt caaaaccaga attccagaaa 181 gatttatctt tttgtaatat tataacaaat attcaaaact ttcaacaacg gatctcttgg 241 ttctcgcatc gatgaagaac gcagcgaaat gcgatacgta atgtgaattg cagaattccg 301 tgaatcatcg aatctttgaa cgcacattgc gccccttg	338	*Kazachstania unispora *IG 16 LC164188.1

*Candida kefyr* 99.9%	1 ttctcatcct aaacacaatg gagttttttc tctatgaact acttccctgg agagctcgtc 61 tctccagtgg acataaacac aaacaatatt ttgtattatg aaaaactatt atactataaa 121 atttaatatt caaaactttc aacaacggat ctcttggttc tcgcatcgat gaagaacgca 181 gcgaattgcg atatgtattg tgaattgcag attttcgtga atcatcaaat ctttgaacgc 241 acattgcgcc ctctggtatt ccagggggca tgcctgtttg agcgtcattt ctctctcaaa 301 cctttgggtt tggtagtgag tgatactcgt ctcgggttaa cttgaaagtg gctagccgtt 361 gccatctgcg tgagcagggc tgcgtgtcaa gtctatggac t	401	*Kluyveromyces marxianus *IG 1 LC126698.1

*Cryptococcus* *albidus* 99.1%	1 ctgacacata cacacagtgg agatatattc tttcttcttc ttcttctttg ggggacggcg 61 gtttgcgctg ctgctcccag aatgaaaaaa cacaaacaac ttttgtatta ttacagatgt 121 caaaacacaa aacaaaatac caaaactttc aacaacggat ctcttggttc tcgcatcgat 181 gaagaacgca gcgaaatgcg atacgtaatg tgaattgcag aattccgtga atcatcgaat 241 ctttgaacgc acattgcgcc ccttggtatt ccagggggca tgcctgtttg agcgtcattt 301 ccttctcaaa cacttgtgtt tggtagtgag tgatactctg taacatggga gttagcttga 361 aattgagagc ccatgggctg ctctgctgag gcggaagtcg tgctaggtaa caccaactcg 421 acgcaacgca ttcggctgga cgcagctccg gcgaacaaac acatcaacgc ttgacctcaa 481 atca	484	*Zygotorulaspora florentina *IG 12

API: Api 20 AUX tests; bp: base pairs.

**Table 2:** Summary of sizes in base pairs of the PCR products and the restriction fragments of yeast DNA.

Strain	PCR-amplified product (bp)	Restriction fragments (bp)									
		*Hae*III					*Rsa*I				
*Candida inconspicua *IG 11	460	290		90		80		360		100	
*Debaryomyces hansenii *1	640	410		140		90		450		190	
*Debaryomyces hansenii *IG II	640	410		140		90		450		190	
*Debaryomyces hansenii *IG 01	640	410		140		90		450		190	
*Kazachstania unispora *IG 16	690		550		130		510		110		70
*Kluyveromyces marxianus *IG 1	700		620		80			630		70	
*Zygotorulaspora florentina *IG 12	600			600				520		80	

bp: base pairs; PCR: polymerase chain reaction.

**Table 3:** Composition of deproteinated potato wastewater used in this study.

Parameter	Unit	Deproteinated potato wastewater
pH		4.9 ± 0.1

Dry substance	g d.w. 100 cm^−3^	3.3260 ± 0.0430

Directly reducing sugars	g 100 cm^−3^	0.4400 ± 0.0032
Nitrogen		0.1620 ± 0.0062
Potassium		0.4137 ± 0.0320
Phosphorus		0.0333 ± 0.0020
Magnesium		0.0236 ± 0.0035
Calcium		0.0100 ± 0.0010

Sodium	mg 100 cm^−3^	6.063 ± 0.051
Manganese		0.181 ± 0.011

**Table 4:** Parameters characterizing the growth and lipid production of kefir yeast strains in media supplemented with glucose, lactose, and glycerol.

Strain	Biomass			*Q*_*x*_			Lipids			*Q*_*L*_				pH	
	Glc	Lac	Gly	Glc	Lac	Gly	Glc	Lac	Gly	Glc	Lac	Gly	Glc	Lac	Gly
	g L^−1^			g L^−1^ h^−1^			g L^−1^			g L^−1^ h^−1^					
	YP medium														
*Candida inconspicua *IG 11	12.2 ± 0.02	13.5 ± 0.3	9.8 ± 0.02	0.127	0.141	0.102	4.04 ± 0.12	3.38 ± 0.11	1.71 ± 0.22	0.042	0.035	0.018	5.10	5.17	5.62
*Debaryomyces hansenii *1	13.5 ± 0.03	12.9 ± 0.2	11.8 ± 0.03	0.140	0.134	0.123	6.31 ± 0.23	2.02 ± 0.09	2.26 ± 0.21	0.066	0.021	0.024	7.31	7.58	6.01
*Debaryomyces hansenii *IG II	13.8 ± 0.01	13.0 ± 0.5	13.6 ± 0.04	0.144	0.135	0.142	1.14 ± 0.03	1.77 ± 0.07	3.28 ± 0.31	0.012	0.018	0.034	6.69	6.97	7.46
*Debaryomyces hansenii *IG 01	14.8 ± 0.02	10.7 ± 0.2	10.2 ± 0.01	0.154	0.111	0.106	5.65 ± 0.15	1.78 ± 0.08	1.85 ± 0.14	0.059	0.018	0.019	7.23	7.69	7.63
*Kazachstania unispora *IG 16	11.0 ± 0.01	10.4 ± 0.4	12.1 ± 0.50	0.114	0.108	0.126	1.70 ± 0.05	1.32 ± 0.09	0.87 ± 0.09	0.018	0.014	0.009	4.29	5.19	6.30
*Kluyveromyces marxianus *IG 1	12.9 ± 0.05	12.2 ± 0.5	10.7 ± 0.50	0.134	0.127	0.111	3.71 ± 0.06	4.76 ± 0.18	0.50 ± 0.09	0.039	0.049	0.005	7.31	7.09	7.03
*Zygotorulaspora florentina *IG 12	13.7 ± 0.04	12.4 ± 0.1	11.3 ± 0.03	0.143	0.129	0.118	4.44 ± 0.07	1.97 ± 0.12	2.34 ± 0.19	0.046	0.020	0.024	5.79	6.31	5.04

	DPW medium														
*Candida inconspicua *IG 11	10.5 ± 0.01	10.9 ± 0.21	10.9 ± 0.02	0.109	0.113	0.113	0.36 ± 0.07	1.58 ± 0.15	2.35 ± 0.11	0.004	0.016	0.024	8.51	8.00	8.01
*Debaryomyces hansenii *1	12.3 ± 0.11	11.6 ± 0.15	11.0 ± 0.03	0.128	0.121	0.112	1.12 ± 0.06	1.99 ± 0.09	1.79 ± 0.09	0.012	0.021	0.018	7.56	7.93	8.44
*Debaryomyces hansenii *IG II	11.7 ± 0.02	12.4 ± 0.02	10.8 ± 0.06	0.122	0.129	0.112	0.85 ± 0.11	0.69 ± 0.02	1.92 ± 0.51	0.009	0.007	0.020	7.30	8.43	8.63
*Debaryomyces hansenii *IG 01	11.4 ± 0.05	11.7 ± 0.05	10.5 ± 0.05	0.119	0.122	0.111	2.80 ± 0.12	2.43 ± 0.16	2.21 ± 0.21	0.030	0.025	0.023	7.10	8.09	8.57
*Kazachstania unispora *IG 16	9.9 ± 0.05	11.6 ± 0.11	10.3 ± 0.11	0.103	0.121	0.107	0.79 ± 0.09	1.91 ± 0.11	2.22 ± 0.16	0.008	0.019	0.023	8.13	8.00	8.01
*Kluyveromyces marxianus *IG 1	10.3 ± 0.02	11.4 ± 0.02	10.7 ± 0.08	0.108	0.119	0.111	3.56 ± 0.12	1.74 ± 0.09	2.16 ± 0.22	0.037	0.018	0.022	7.94	8.52	8.42
*Zygotorulaspora florentina *IG 12	10.1 ± 0.03	11.9 ± 0.50	11.1 ± 0.08	0.105	0.123	0.115	0.52 ± 0.21	0.85 ± 0.07	1.97 ± 0.19	0.005	0.009	0.020	7.13	8.13	8.28

The final concentration of biomass, the volumetric productivities of biomass (*Q*_*x*_), lipids, and volumetric productivities of lipids (*Q*_*L*_). The values are mean of three independent experiments.

**Table 5:** Relative fatty acid composition (%) in kefir yeast biomass after culturing in medium supplemented with glucose (YPD medium).

Strain	Relative fatty acids composition (%)														
	C12:0	C14:0	C16:0	C16:1	C17:1	C18:0	C18:1	C18:2	C18:3 n-3	C20:1 n-9	C20:3 n-6	C20:4 n-6	C22:2	C22:4	C24:0
*Candida inconspicua* IG11	n.d.	n.d.	16.61^c^	2.18^a^	n.d.	12.28^c^	48.5^c^	18.28^c^	2.15^b,c^	n.d.	n.d.	n.d.	n.d.	n.d.	n.d.
*Debaryomyces hansenii* 1	n.d.	n.d.	15.94^c^	3,80^b^	n.d.	11,50^c^	46,20^d^	15,21^c^	2.53^a,b^	0.89^a^	n.d.	n.d.	n.d.	3,93^b^	n.d.
*Debaryomyces hansenii* IG II	0.33^a^	n.d.	17.85^c^	3.79^b^	n.d.	12.03^c^	37.69^b^	14.18^b^	1.42^b^	0.92^a^	n.d.	n.d.	n.d.	4.97^b^	6.82^b^
*Debaryomyces hansenii* IG 01	n.d.	n.d.	14.04^b^	1.80^a^	n.d.	11.23^c^	60.25^d^	8.68^a^	n.d.	0.92^a^	n.d.	n.d.	n.d.	n.d.	3.08^a^
*Kazachstania unispora* IG 16	n.d.	2.37^b^	8.31^a^	39.61^d^	n.d.	2.09^a^	20.83^a^	n.d.	1.92^b^	n.d.	5.47^a^	8.14^c^	2.09^a^	n.d.	9.17^c^
*Kluyveromyces marxianus* IG 1	n.d.	n.d.	14.95^b^	10.61^c^	n.d.	5.21^b^	34.87^b^	10.66^a^	1.78^b^	2.54^b^	n.d.	6.28^b^	n.d.	n.d.	13.1^d^
*Zygotorulaspora florentina* IG 12	n.d.	0.65^a^	17.21^c^	9.61^c^	0.49^a^	8.28^b,c^	43.39^b,c^	10.48^a^	0.54^a^	n.d.	n.d.	2.11^a^	3.18^a^	n.d.	4.06^a^

n.d. = not detected. a, b, c mean values marked with the same letters do not differ significantly. Tukey's test *α* = 0.05. *Note*. Samples were taken on third or fourth day of cultivation (statistically no differences). Fatty acid content is given as mean value of three independent experiments. Standard deviation was less than 5%.

**Table 6:** Yeast strains isolated from kefir and kefir grains: a literature review.

Strain	References
*Brettanomyces anomalus*	[19–21]

*Candida famata* *Candida firmetaria*	[21]

*Candida friedrichii*	[20, 21]

*Candida holmii*	[19, 20, 22]

*Candida humilis*	[21]

*Candida inconspicua*	[20, 21, 23]

*Candida kefyr*	[19–24]

*Candida krusei*	[21, 25, 26]

*Candida lambica*	[19, 20, 25]

*Candida lipolytica*	[19, 21]

*Candida maris*	[20, 21, 23]

*Candida tannotelerans*	[20]

*Candida tenuis* *Candida valida*	[19, 20]

*Cryptococcus humicolus*	[21, 25]

*Debaryomyces hansenii* *Dekkera anomala* *Galactomyces geotrichum*	[21]

*Geotrichum candidum*	[19, 21, 25]

*Issatchenkia occidentalis*	[19, 20]

*Issatchenkia orientalis*	[21, 26]

*Kazachstania aerobia* *Kazachstania salicola* *Kazachstania serovazzii Kazachstania turicensis* *Kazachstania unispora*	[27]

*Kluyveromyces bulgaricus* *Kluyveromyces fragilis*	[19]

*Kluyveromyces lactis *var. *lactis*	[19, 21, 23, 26]

*Kluyveromyces lodderae*	[21]

*Kluyveromyces marxianus*	[19–22]

*Kluyveromyces marxianus* var. *lactis*	[23]

*Pichia fermentans*	[19–21, 23]

*Saccharomyces cerevisiae*	[19–24]

*Saccharomyces dairensis*	[20]

*Saccharomyces delbrueckii*	[19, 20]

*Saccharomyces exiguous*	[19–22, 26]

*Saccharomyces fragilis*	[24]

*Saccharomyces humaticus*	[26]

*Saccharomyces lactis*	[24]

*Saccharomyces pastorianus*	[21]

*Saccharomyces turicensis *	[20, 21]

*Saccharomyces unisporus*	[19–21, 23, 26]

*Schwanniomyces* *occidentalis*	[21]

*Torulaspora* *delbrueckii*	[19–21, 23]

*Yarrowia lipolytica*	[19, 21, 23]

*Zygosaccharomyces* *florentinus*	[19]

*Zygosaccharomyces rouxii*	[21]

*Zygosaccharomyces *sp.	[25]

**Table 7:** Biodiesel properties based on the fatty acids composition of the microbial oil producers by kefir yeast strain isolates in different media.

Strain	Carbon source	Nitrogen source	UD	CN	LC	LCV (kJ kg^−1^)	FP (°C)	*µ* (mm^2^ s^−1^)
*Candida inconspicua* IG 11	Glucose	Peptone	0,94	58	17,61	35595,71	172,41	5,32
		DPW	1,04	53	17,65	37575,43	170,98	5,19
	Lactose	Peptone	0,77	61	17,40	37560,82	170,06	5,45
		DPW	1,00	54	17,68	37604,87	173,07	5,27
	Glycerol	Peptone	0,84	59	17,49	37573,25	170,86	5,39
		DPW	1,03	54	17,69	37598,66	172,62	5,24

*Debaryomyces hansenii *1	Glucose	Peptone	0,88	55	18,01	37813,79	189,27	5,67
		DPW	0,99	56	17,61	37576,05	170,99	5,25
	Lactose	Peptone	0,65	65	17,45	37630,43	175,44	5,68
		DPW	0,70	59	17,19	37474,17	164,08	5,38
	Glycerol	Peptone	0,74	58	17,44	37587,95	172,06	5,52
		DPW	0,91	56	17,61	37559,11	169,86	5,18

*Debaryomyces hansenii *IG II	Glucose	Peptone	0,75	53	17,31	37522,23	167,36	5,41
		DPW	0,88	55	17,49	37559,45	169,84	5,33
	Lactose	Peptone	0,86	51	18,21	37917,75	198,08	5,84
		DPW	0,92	57	17,51	37553,68	169,41	5,28
	Glycerol	Peptone	0,75	57	17,24	37488,15	165,00	5,37
		DPW	0,92	59	17,41	37504,99	166,04	5,23

*Debaryomyces hansenii *IG 01	Glucose	Peptone	0,80	58	17,89	37782,18	186,87	5,72
		DPW	0,92	58	17,58	37585,46	171,68	5,33
	Lactose	Peptone	0,86	51	15,53	37585,35	171,71	5,39
		DPW	0,92	57	17,47	37535,54	168,15	5,26
	Glycerol	Peptone	0,75	40	16,19	36976,93	135,68	4,69
		DPW	0,92	58	17,32	37461,17	163,09	5,16

*Kazachstania unispora* IG16	Glucose	Peptone	0,70	41	17,85	37804,41	188,96	5,86
		DPW	0,85	57	16,55	37118,81	142,77	4,79
	Lactose	Peptone	0,77	59	17,34	37531,19	167,96	5,41
		DPW	1,05	52	17,00	37256,05	150,61	4,79
	Glycerol	Peptone	0,90	56	17,02	37323,10	154,32	5,00
		DPW	1,06	51	16,99	37100,16	142,32	4,59

*Kluyveromyces marxianus *IG 1	Glucose	Peptone	0,75	45	18,19	37951,52	201,39	6,02
		DPW	0,89	56	17,35	37488,00	164,89	5,23
	Lactose	Peptone	0,76	46	18,11	37907,84	197,52	5,94
		DPW	0,85	59	17,25	37453,83	162,61	5,23
	Glycerol	Peptone	0,85	59	17,43	37542,21	168,65	5,34
		DPW	0,91	57	17,51	37557,56	169,69	5,30

*Zygotorulaspora florentina *IG 12	Glucose	Peptone	0,82	54	17,85	37754,23	184,57	5,66
		DPW	0,85	59	17,45	37554,12	169,49	5,36
	Lactose	Peptone	0,67	55	16,12	36979,07	135,56	4,76
		DPW	0,81	61	17,38	37535,33	168,20	5,37
	Glycerol	Peptone	0,86	58	17,50	37570,52	170,64	5,36
		DPW	0,91	64	17,55	37577,03	171,08	5,33

Unsaturation degree (UN), cetane number (CN), length of chain (LC), low caloric value (LCV), flash point (FP), and viscosity (*μ*).
