# Supplementary material for: Identification and Characterization of Oleaginous Yeast Isolated from Kefir and Its Ability to Accumulate Intracellular Fats in Deproteinated Potato Wastewater with Different Carbon Sources
Source: Biomed Res Int. 2017 Sep 17;2017:6061042. doi: 10.1155/2017/6061042 (PMC5623792; doi:10.1155/2017/6061042)

Table S1. Dimensions of the cells of kefir yeast strains after the culture in YPD medium.

**Strain**	**Length [µm]**	**Width [µm]**	**Area [µm^2]**
Candida inconspicua IG 11	3.04 ± 0.72	1.86 ± 0.32	4.45 ± 1.62
Debaryomyces hansenii I	1.84 ± 0.45	1.80 ± 0.45	2.88 ± 1.22
Debaryomyces hansenii IG II	1.79 ± 0.35	1.75 ± 0.45	2.47 ± 1.20
Debaryomyces hansenii IG 01	2.03 ± 0.34	1.99 ± 0.35	3.42 ± 1.04
Kazachstania unispora IG 16	2.42 ± 0.51	1.89 ± 0.38	3.30 ± 1.24
Kluyveromyces marxianus IG 1	2.90 ± 0.64	1.68 ± 0.32	3.87 ± 1.39
Zygotorulaspora florentina IG 12	2.46 ± 0.47	2.27 ± 0.42	4.75 ± 1.42

Table S2. Maximum growth rates (μ_{max}) of kefir yeast strain during incubation in Bioscreen C.

Strain	YPGlu	YPLac	YPGly	DPWGlu	DPWLac	DPWGly
	μ_{max} (h^{-1})					
Candida inconspicua IG 11	$0.200 \pm 0.05^{b.c}$	0.061 ± 0.01^{b}	0.078 ± 0.01^{b}	0.098 ± 0.08^{c}	0.037 ± 0.06^{a}	0.045 ± 0.02^{a}
Debaryomyces hansenii 1	0.137 ± 0.04^{b}	0.071 ± 0.02^{b}	0.082 ± 0.03^{b}	0.088 ± 0.02^{b}	0.051 ± 0.02^{b}	0.094 ± 0.04^{c}
Debaryomyces hansenii IG II	0.130 ± 0.05^{b}	$0.095 \pm 0.03^{b.c}$	0.047 ± 0.03^{a}	0.038 ± 0.04^{a}	0.029 ± 0.02^{a}	0.021 ± 0.01^{a}
Debaryomyces hansenii IG 01	0.152 ± 0.04^{b}	0.050 ± 0.02^{a}	0.045 ± 0.01^{a}	0.033 ± 0.02^{a}	0.027 ± 0.01^{a}	0.054 ± 0.02^{b}
Kazachstania unispora IG 16	0.072 ± 0.01^{a}	0.064 ± 0.01^{b}	$0.063 \pm 0.02^{a.b}$	0.081 ± 0.02^{b}	0.074 ± 0.01^{c}	0.037 ± 0.03^{a}
Kluyveromyces marxianus IG 1	0.077 ± 0.02^{a}	0.069 ± 0.03^{b}	$0.045 \pm 0\ .02^{a}$	0.067 ± 0.03^{b}	0.062 ± 0.02^{b}	0.064 ± 0.02^{b}
Zygotorulaspora florentina IG 12	0.190 ± 0.05^{c}	0.036 ± 0.02^{a}	0.030 ± 0.03^{a}	0.034 ± 0.01^{a}	0.030 ± 0.01^{a}	0.022 ± 0.01^{a}

YPGlu: Yeast peptone agar supplemented with glucose; YPLac: Yeast peptone agar supplemented with lactose; YPGly: Yeast peptone agar supplemented with glycerol; DPWGlu: deproteinated potato wastewater supplemented with glucose; DPWLac: deproteinated potato wastewater supplemented with lactose; DPWGly: deproteinated potato wastewater supplemented with glycerol.

Fig. S1 Phylogenetic analysis of yeast isolates.

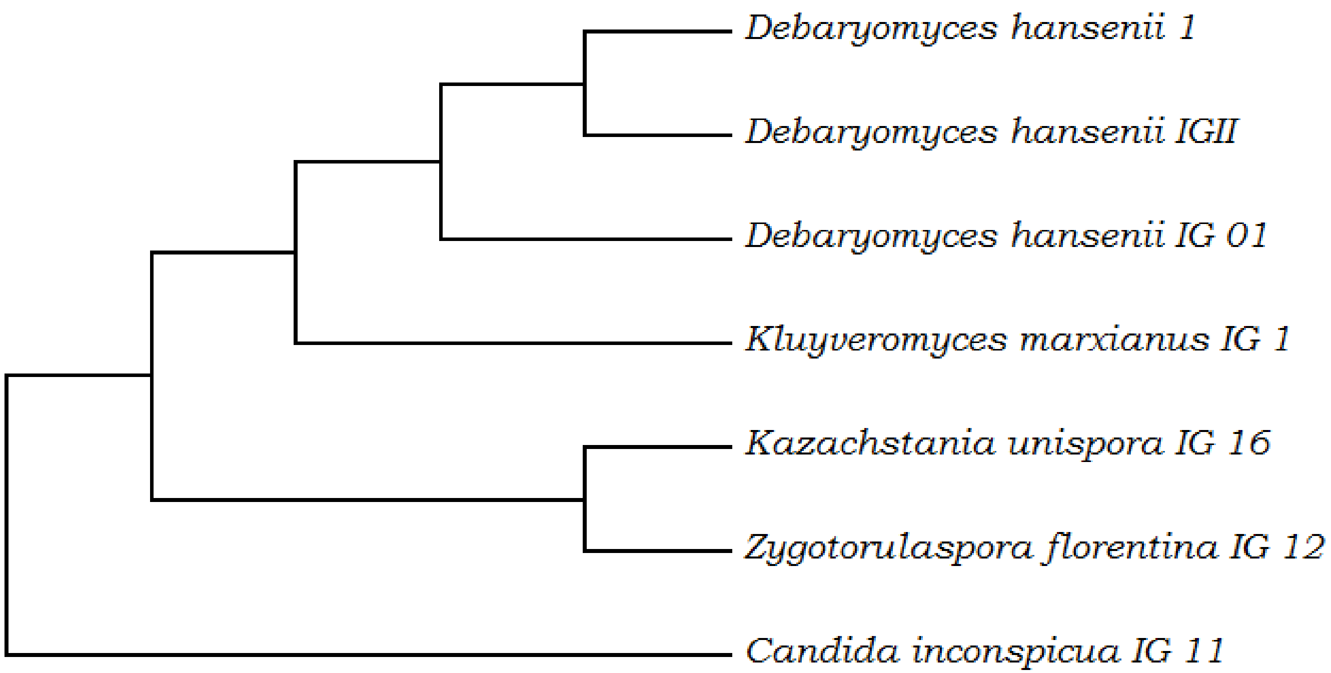

Fig. S2 Electrophoretic separation of proteins of deproteinated potato wastewater.

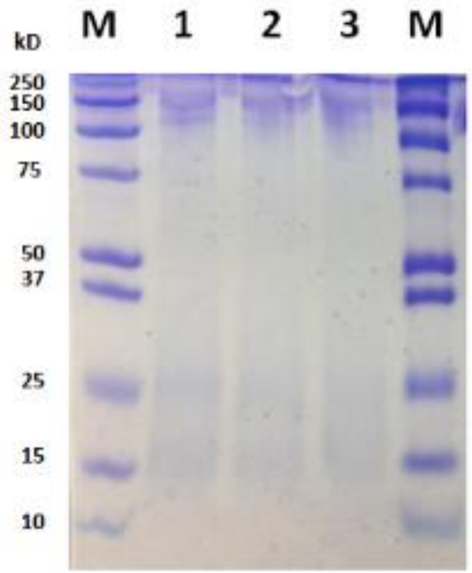

Supplement: Supplementary file 1 — Supplementary materials contain information about dimensions of the cells of kefir yeast strains and its maximum growth rates. [file 6061042.f1.pdf]
